# Cell-Penetrating Botulinum Neurotoxin Type A With Improved Cellular Uptake and Therapeutic Index

**DOI:** 10.3389/fbioe.2022.828427

**Published:** 2022-02-11

**Authors:** Xuan Wei, Lu Li, Yiwen Wu, Jia Liu

**Affiliations:** ^1^ Shanghai Institute for Advanced Immunochemical Studies and School of Life Science and Technology, ShanghaiTech University, Shanghai, China; ^2^ Department of Neurology & Institute of Neurology, Ruijin Hospital, Shanghai Jiao Tong University School of Medicine, Shanghai, China; ^3^ Shanghai Clinical Research and Trial Center, Shanghai, China

**Keywords:** cell-penetrating peptides, zinc finger proteins, botulinum neurotoxin type A, cellular uptake, therapeutic index

## Abstract

Botulinum neurotoxin serotype A (BoNTA) is widely used for treating neuromuscular disorders. Despite of the various marketed products, BoNTA is known to have small therapeutic index ranging from 5 to 15. In the present study, we designed and characterized engineered BoNTA proteins with fusion of cell-penetrating peptides (CPPs). We have shown that CPPs, particularly a recently reported zinc finger protein could improve the cellular uptake and intramuscular therapeutic index of BoNTA. Our study has shed the light on developing next-generation neuromuscular modulators using CPP fusion.

## Introduction

Botulinum neurotoxins (BoNTs) are neurotoxic proteins produced by *Clostridium botulinum* and related bacterial species (Rossetto et al., 2014). BoNTs contain seven major serotypes (A-G), but all have common structural features and a similar mechanism of action. Most of the naturally occurring BoNTs are produced by bacteria as single polypeptide chains and then cleaved into a 100 kDa heavy chain (HC) and a 50 kDa light chain (LC), which are connected through a single disulfide bond (Dong et al., 2006). In the case of type A BoNT (BoNTA), the HC domain binds to the SV2 receptor on motor nerve terminals which mediates the cellular uptake of BoNTA (Dong et al., 2006). The LC domain of BoNTA specifically cleaves the 25-kD synaptosomal nerve-associated protein (SNAP-25) that is in charge of the docking and fusion of cellular vesicles (Jahn and Scheller, 2006).

Small doses of BoNTs can be applied to block neuromuscular connections and thereby effect targeted therapeutic paralysis of muscle groups. BoNTA-containing protein complexes ona-, abo- and incobotulinum have been developed as commercial products under the trade names of Botox, Dysport, and Xeomin, respectively. These products are widely used as therapeutic and cosmetic agents and have been approved for treating a wide range of neuromuscular disorders. Marketed BoNTA products primarily differ in purity, stability and excipients (Dressler, 2012). While the overall patient satisfaction of injectable BoNTA is above 80%, an increasing number of severe and long-term adverse effects have been observed with the constantly expanding medical indications (Lim and Seet, 2010). These adverse effects are largely due to the suboptimal diffusion of BoNTA and its narrow therapeutic index of 5–15 (Aoki, 2001; Comella et al., 2005; Rossetto et al., 2014). The narrow therapeutic index of BoNTA also impede the formulation of BoNTA products and its distribution in the market, where the maximum dose per vial and the number of purchased vials are strictly regulated. In order to overcome the limitations on small therapeutic index and drug safety, engineering endeavors are needed to improve the pharmacological properties of BoNTA proteins.

Cell-penetrating peptides (CPPs) represents a unique and compelling opportunity for protein delivery along with other approaches such as lipoparticles and viral vectors. CPPs are short peptides that can deliver myriad biomolecules including peptides, proteins and nucleic acids into cells (Khan et al., 2020). We previously described a zinc finger protein (ZFP)-based CPP that allowed for efficient intracellular delivery of diverse proteins (Gaj et al., 2012; Gaj et al., 2014; Gaj and Liu, 2015; Liu et al., 2015; Liu et al., 2018; Pang et al., 2019). ZFPs penetrate cells through macropinocytosis and caveolin-dependent endocytosis pathways and have high efficiency of endosome escape. Importantly, ZFPs have been shown to outperform traditional CPPs and are capable of delivering cargo proteins to a wide range of cell types including primary cells (Gaj et al., 2014; Liu et al., 2015). In the present study, we proposed that genetic fusion of ZFPs as well as other CPPs such as Pep1 (Morris et al., 2001) or TAT (Fawell et al., 1994) can enhance the cellular uptake of BoNTA and improve its pharmacological properties. We designed a series of CPP-BoNTA proteins with different architectures and evaluated their *in vitro* and *in vivo* activity and toxicity.

## Materials and Methods

### Protein Expression and Purification

BoNTA gene was synthesized by GENEWIZ Inc. (Nanjing, Jiangsu, China) and codon optimized for expression in *Spodoptera frugiperda* Sf9 cells as described (Band et al., 2010). His_6_ tag and GS linker were added into the fusion genes and the constructs were verified by Sanger sequencing. CPP-BoNTA coding sequence was transferred from pFastBac to bacmid by transposition in DH10Bac *Escherichia coli* according to manufacturer’s instructions of the Bac-to-Bac baculovirus expression system (Invitrogen, Carlsbad, California, United States). Recombinant bacmids were isolated and purified from *E. coli* cells using QIAGEN Large Construct Kit (QIAGEN, Germantown, Maryland, United States) according to manufacturer’s protocol. After three passages of virus amplification, a virus stock solution with high virus titer was obtained. After virus transduction and protein expression, the insect cell lysate was purified by nickel-nitrilotriacetic acid (Ni-NTA) Sepharose affinity resin (QIAGEN). The proteins were further purified using fast protein liquid chromatography with Superdex 200 Increase 10/300 GL column (GE Healthcare, Shanghai, China). The purified proteins were harvested and stored at −80°C.

### *In vitro* Peptide Cleavage Assay

The *in vitro* peptide cleavage assay was based on fluorescence resonance energy transfer (FRET) as previously described (Baldwin et al., 2004). The peptide substrate contains target sequence that is derived from the native BoNTA substrate, SNAP-25. In the present study, we synthesized a substrate peptide with the sequence FITC-Thr-(D-Arg)-Ile-Asp-Gln-Ala-Asn-Gln-Arg-Ala-Thr-Lys-(DABCYL)-Nle-NH2 (GL Biochem Corporation, Shanghai, China). In this peptide, the N-terminal fluorophore is fluorescein isothiocyanate (FITC) and C-terminal quencher is 4-((4-(dimethylamino) phenyl) azo) benzoic acid (DABCYL). Upon cleavage of the peptide, the fluorophore FITC will be released and the activated fluorescence signal can be measured spectroscopically. Characterization of the FRET peptide can be found in the supplementary information. The cleavage reaction contained 20 mM HEPES, pH 7.4, 0.05% Tween 20, 100 nM recombinant CPP-BoNTA and 10 μM SNAPtide substrate and was incubated at 37°C for 40 min. The fluorescence was measured by a plate reader with an excitation wavelength of 490 nm and an emission wavelength of 523 nm.

### Cell Culture

Mouse neuroblastoma N2a cells were maintained in DMEM (Gibco) supplemented with 10% FBS (Gibco), 1% non-essential amino acids (Gibco) and 100 U ml^−1^ penicillin/streptomycin (Gibco) at 37°C in fully humidified atmosphere with 5% CO_2_.

### Cytotoxicity Assay

The *in vitro* cytotoxicity of BoNTA proteins was determined on mouse N2a cells using Cell Counting Kit-8 (CCK-8) (Dojindo) according to manufacturer’s protocol. Briefly, 1 × 10^3^ cells were seeded on to 96-well plates. At 24 h after seeding, the cells were treated with 100 nM WT- and CPP-BoNTA proteins in serum-free DMEM medium. After 2 h transduction, the cells were supplemented with 100 μL fresh DMEM medium containing 10 μL CCK8 solution. The cell culture was incubated at 37°C for 2 h and the absorbance was measured at 450 nm.

### Experimental Animals

All experiments were conducted in accordance with the guidelines of the American Association for the Accreditation of Laboratory Animal Care (AAALAC). All animal experimentation was conducted in accordance with the regulations of Animal Care and Use Committee, Shanghai Model Organisms Center, Inc. Eight week old, C57BL/6J female mice (17–22 g, Shanghai Model Organisms Center, Shanghai, China) were housed in a barrier facility and were maintained on a 12-h light/dark cycle (7 a.m.–7 p.m.) with *ad libitum* access to food and water.

### Injection Procedure

Each mouse received intramuscular injection of CPP-BoNTA or vehicle into the head of the right gastrocnemius muscle. Injections were made in a fixed volume of 5 μL using a 30-gauge needle attached to a sterile 250 μL Hamilton syringe. For each experiment, at least five mice were injected per dose.

### Immunofluorescence Staining

Mouse neuroblastoma N2a cells were seeded on coverslips in culture dishes and grown to a confluency of 70–80%. Cells were then fixed using 4% paraformaldehyde (BBI Life Sciences Corporation, Shanghai, China) and permeabilized with phosphate buffered saline (PBS) containing 0.1% Triton X-100 for 10 min. Cells were blocked using blocking solution containing PBS supplemented with 3% bovine serum albumin (BSA) (Solarbio Life Sciences, Beijing, China) and incubated overnight with goat anti-FLAG (Novus Biologicals, Littleton, Colorado, United States) and rabbit anti-SV2A antibodies (Novus) at 1 to 200 dilution in PBS supplemented with 0.2% BSA. Cells were then washed with PBS supplemented with 0.2% BSA and incubated with Alexa568-conjugated donkey anti-rabbit IgG (Invitrogen) and Alexa488-conjugated donkey anti-goat IgG (Invitrogen) secondary antibodies.

The treated gastrocnemius muscles were sectioned, immediately fixed with 4% paraformaldehyde and dehydrated overnight in 30% sucrose. The tissue blocks were then dried on paper towel and placed on tissue molds that were sequentially filled with 100% optimal cutting temperature compound (OCT) over a total period of 4 h at −80°C. OCT-embedded gastrocnemius muscles were serially frozen-sectioned at 10 μm interval along the horizontal direction. Section slices were blocked using blocking solution containing PBS and 5% FBS (Solarbio), then incubated with anti-FLAG antibody (Novus) and anti-SV2A antibodies (Novus), washed with PBS and incubated with secondary antibodies.

Antibody-labeled cells and tissue sections were stained with Hoechst 33,342 (Invitrogen) for nucleus visualization. Images were obtained using LSM710 laser scanning confocal microscopy (Carl Zeiss Microscopy GmbH, Jena, Germany) and TissueFAXS (TissueGnostics, Vienna, Austria) fluorescence imaging system. For confocal microscopy, the excitation/emission filters for red and green channels are 493/598 nm and 410/507 nm respectively. The fluorescence intensity in each cell was measured by ZEN 2011imaging software (Zeiss). For the TissueFAXS, the whole section slices were scanned and fluorescence intensity was calculated using TissueQuest software (TissueGnostics).

### Digit Abduction Score (DAS) Assay

The mouse DAS assay was used to determine the pharmacologic activity of BoNTA preparations by measuring the muscle-weakening effectiveness (Aoki, 2001). In the DAS assay, mice were briefly suspended by their tails to elicit a characteristic startle response in which the mice extended their hind-limbs and abducted their hind digits. Following BoNTA injection, the degrees of digit abduction were scored on a five-point scale by two separate observers, with greater scores indicating more muscle-weakening effects. The peak DAS response at each dose, which was typically observed on Day 2 or 3 post injection, was fitted into linear or logarithmic regression equations for calculations of the half effective dose by intramuscular injection (IMED_50_). The IMED_50_ value was defined as the dose at which half of the mice produced a DAS value of 2 (Aoki, 2001).

### Determination of Systemic Effects and Therapeutic Index by Intramuscular Injection

The half lethal dose by intramuscular injection (IMLD_50_) was defined as the dose at which half of the mice died following treatment. The end point of monitoring was set at day 5, after which no further death was found. This lethality reflects the systemic effects of BoNTA considering neurotoxin escape from the muscle and its circulation through the whole body. The intramuscular therapeutic index, or margin of safety, of each sample was defined as the ratio between IMLD_50_ and DAS IMED_50_ values that were obtained from the same experiment.

## Results

### Design and Characterization of CPP-BoNTA Proteins

**FIGURE 1:**
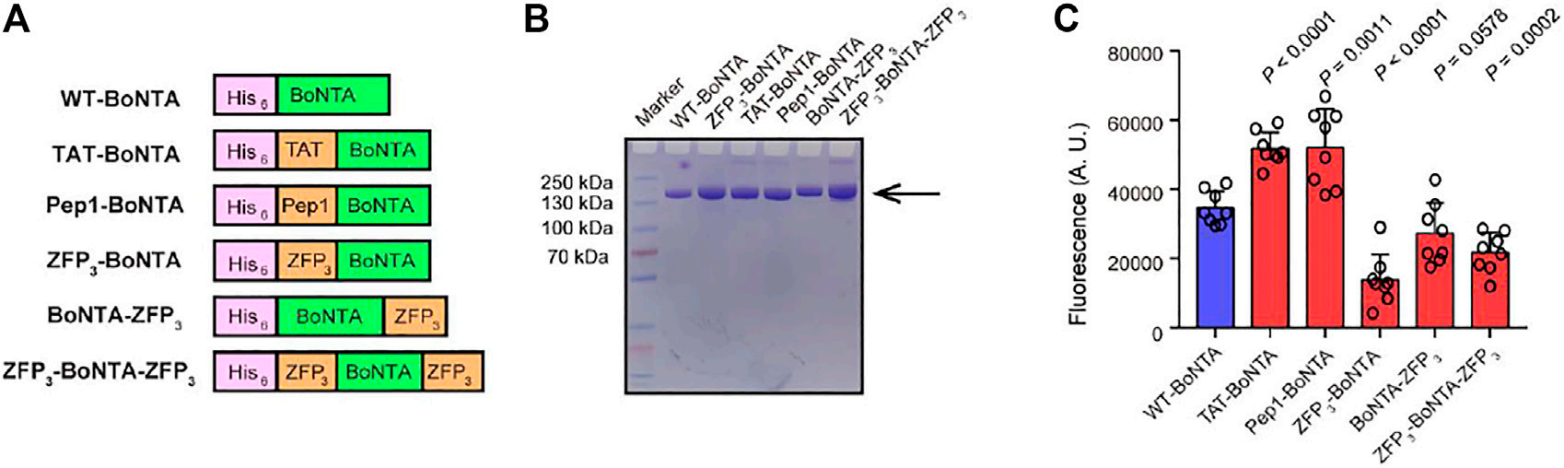
Design and characterization of CPP-BoNTA proteins. **(A)** Schematic presentation showing the design of CPP-BoNTA fusion proteins. **(B)** Reducing SDS-PAGE gel showing purified CPP-BoNTA proteins. Arrow denotes target protein bands. **(C)** *In vitro* activity of CPP-BoNTA, as determined by the cleavage of a reporter peptide. Data are shown as mean ± standard deviation (SD; *n* = 8). Significant difference between WT and CPP-BoNTA is determined using Student’s *t* test.

In this study, we designed BoNTA proteins with different CPP fusion including TAT, Pep1, N-terminal ZFP, C-terminal ZFP and bipartite ZFPs. All protein constructs contain His_6_ tags for affinity purification (Figure 1A). Wild-type and CPP-BoNTA proteins were expressed in insect cells using baculovirus (BV) expression system and were purified to more than 95% homogeneity with yields ranging from 5 to 30 mg per liter culture (Figure 1B). We sought to characterize the *in vitro* protease activity of CPP-BoNTA using a fluorescence resonance energy transfer (FRET) peptide reporter (Supplementary Figure S1). We found that CPP fusion affected the cleavage activity of BoNTA by different manners and degrees (Figure 1C).

### Evaluation of the Cellular Uptake of CPP-BoNTA Proteins *In Vitro*

**FIGURE 2:**
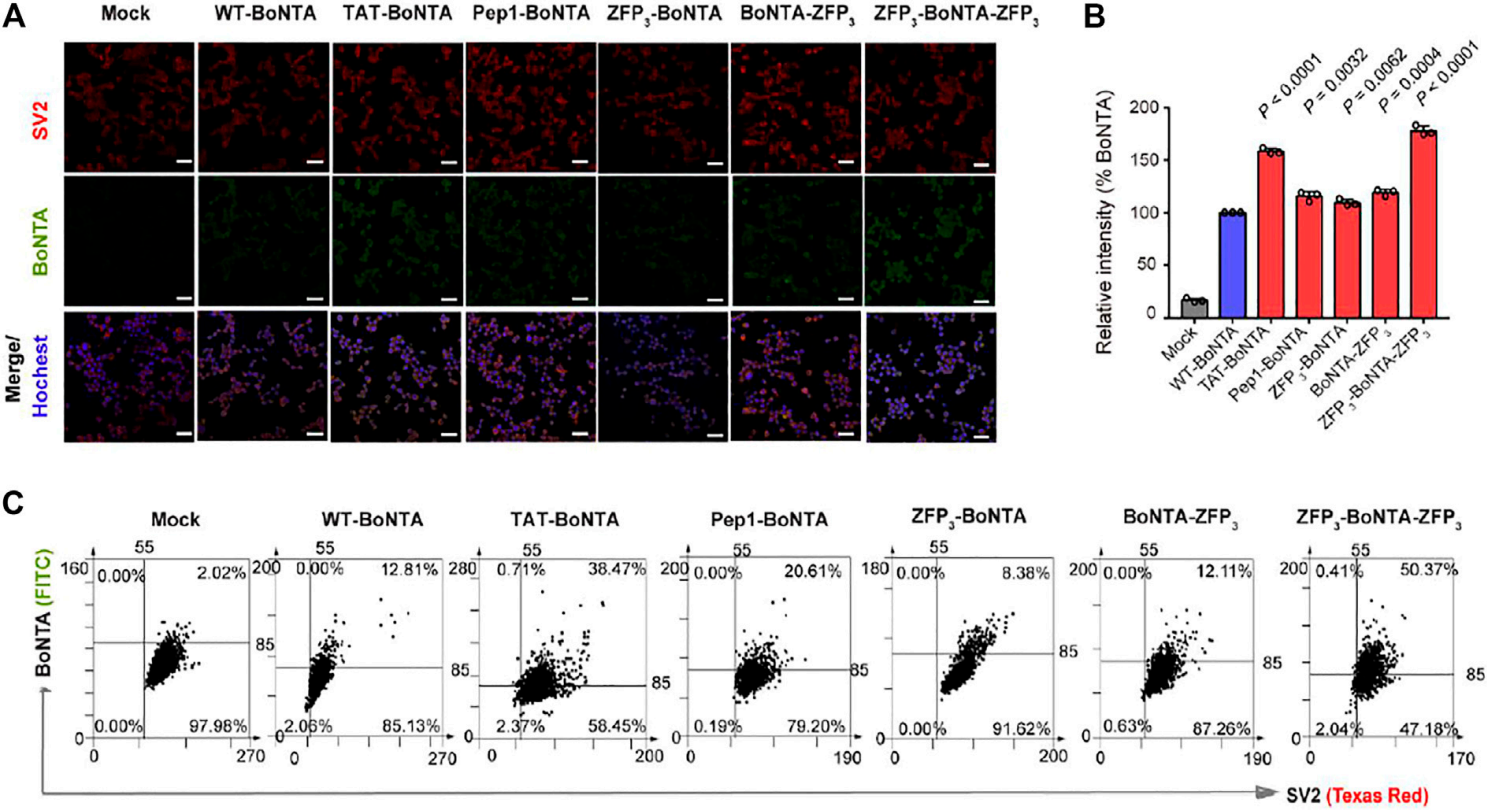
Evaluation of the cell-penetrating activity of CPP-BoNTA in mouse N2a cells with 2 h incubation. **(A,B)** Immunofluorescence (IF) analysis of the cell-penetrating activity of CPP-BoNTA. **(A)** Representative images. Scale bars, 20 μm. **(B)** Quantification of the mean fluorescence intensity of BoNTA positive cells. The data are shown as mean ± SD (*n* = 3). Statistical analysis is performed using Student’s *t* test. **(C)** TissueFAXS cytometry analysis of co-localization of BoNTA proteins and SV2 receptors in mouse N2a cells. Three biological replicates are performed and 1,000 cells are analyzed for each replicate.

We first analyzed the cytotoxicity of BoNTA proteins in mouse neuroblastoma N2a cells. N2a cells were grown to a confluency of 70–80% and then incubated with 100 nM WT- and CPP-BoNTA proteins for 2 h at 37°C. It was found that CPP fusion had minor or no effects on the *in vitro* cytotoxicity of BoNTA (Supplementary Figure S2A). We then analyzed the cell-penetrating activity of BoNTA proteins. It was found that different CPPs enhanced the cellular uptake of BoNTA by various degrees, with TAT and bipartite ZFP fusions exhibiting highest improvement, as determined by immunofluorescence (Figures 2A,B). Prolonged incubation of N2a cells with BoNTA proteins for 4 h showed that TAT-BoNTA and ZFP_3_-BoNTA-ZFP_3_ consistently displayed highest improvement of cellular uptake (Supplementary Figures S2B,C).

Importantly, both WT- and CPP-BoNTA were co-localized with BoNTA receptor SV2 (Figure 2C; Supplementary Figure S2D,E; Benoit et al., 2014), suggesting that CPP fusion did not alter the dependency of SV2 for BoNTA internalization. It seemed that the effects of CPP fusion on the *in vitro* cleavage and cell-penetrating activities of BoNTA were not necessarily related (Figures 1C, 2B). In addition, although immunofluorescence and TissueFAX analyses exhibited variations of the relative cell-penetrating activities among different BoNTA proteins (Figures 2B,C), ZFP_3_-BoNTA-ZFP_3_ consistently exhibited highest cellular uptake efficiency.

### Evaluation of the Cellular Uptake of CPP-BoNTA Proteins *In Vivo*

**FIGURE 3:**
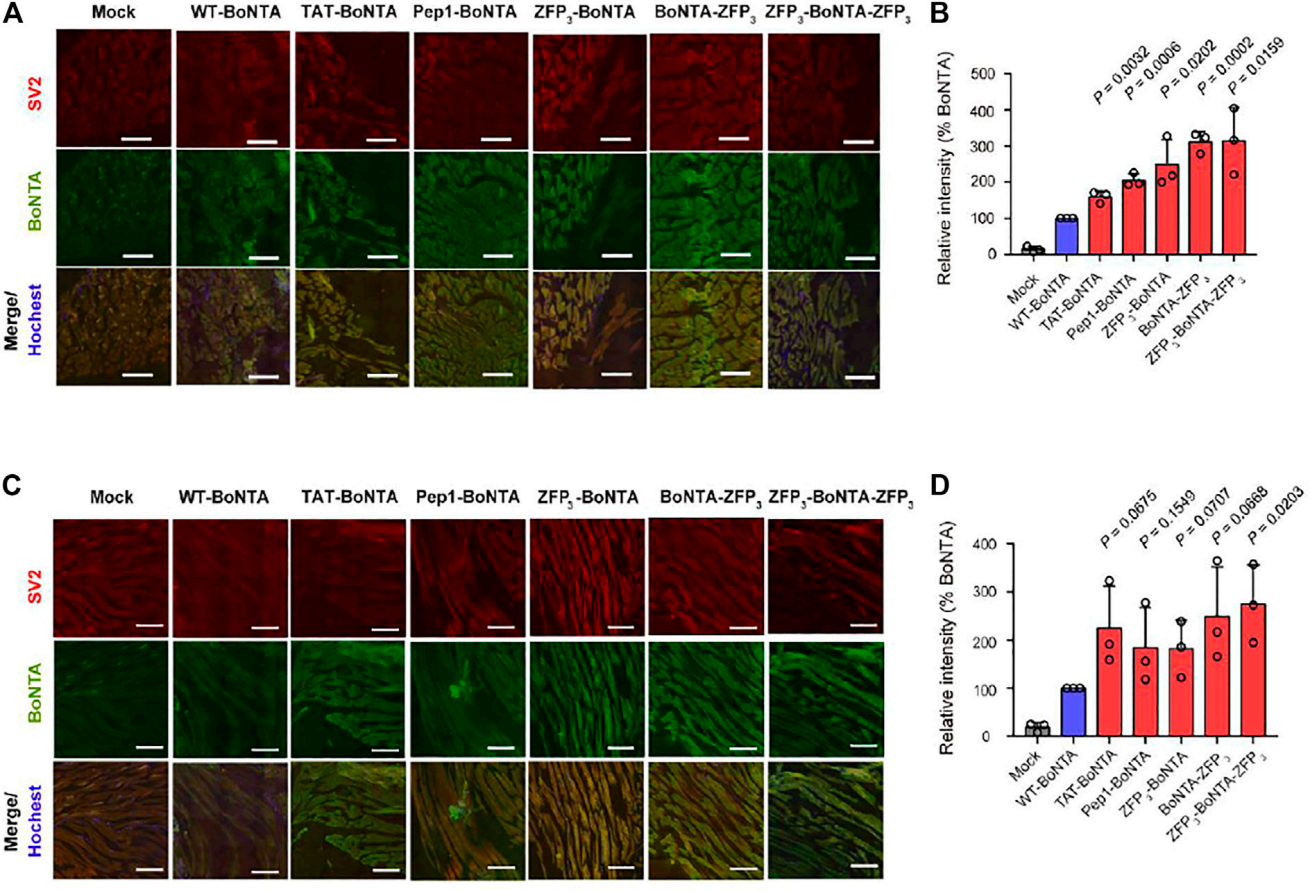
*In vivo* cellular uptake of intramuscularly injected CPP-BoNTA in mouse gastrocnemius muscles. **(A)** Representative images of transverse sections. Scale bars, 200 μm. **(B)** Quantification of the mean fluorescence intensity of BoNTA positive cells in **A**. **(C)** Representative images of longitudinal sections. Scale bars, 200 μm. **(D)** Quantification of the mean fluorescence intensity of BoNTA positive cells in **C**. **(B,D)** The significant difference between WT and CPP-BoNTA is determined using Student’s *t* test.

We injected CPP-BoNTA proteins to the gastrocnemius muscles of mice and evaluated their *in vivo* cell-penetrating activity. Consistent with the *in vitro* results, CPP fusion significantly enhanced the cellular uptake of BoNTA in gastrocnemius muscles with C-terminal and bipartite ZFP fusions displaying highest efficiency, regardless of the directions of sections (Figure 3).

### Evaluation of the Systemic Toxicity of CPP-BoNTA Proteins

**FIGURE 4:**
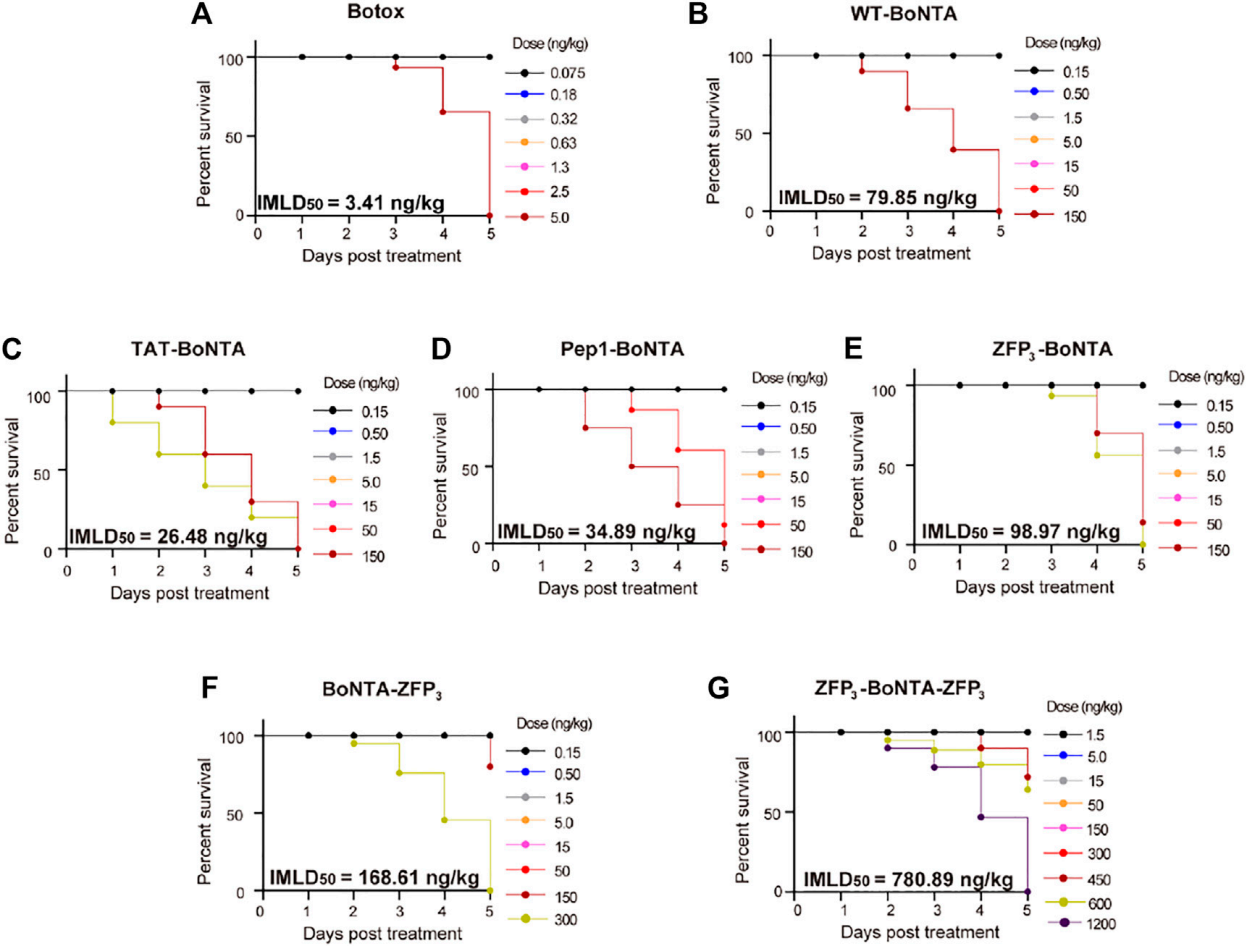
Evaluation of the systemic toxicity of intramuscularly injected BoNTA in mice. Dose-dependent acute toxicity of **(A)** Botox **(B)** BoNTA **(C)** TAT-BoNTA **(D)** Pep1-BoNTA **(E)** ZFP_3_-BoNTA **(F)** BoNTA-ZFP_3_ **(G)** ZFP_3_-BoNTA-ZFP_3_ at the end point of monitoring (day 5 after treatment).

We sought to examine the intramuscular toxicity of CPP-BoNTA in mice (Figures 4A–G). It was found that all recombinant BoNTA derived from insect cells had lower intramuscular toxicity (higher IMLD_50_) than Botox. Importantly, all ZFP fusions could reduce the toxicity of BoNTA, with bipartite ZFP fusion (ZFP_3_-BoNTA-ZFP_3_) displaying greatest decrease in toxicity (Figure 4). By contrast, TAT- and Pep1-BoNTA showed increased toxicity in comparison with WT-BoNTA (Figures 4C,D). Following conventional standard to define BoNTA potency using systemic lethality, herein we defined one active unit of WT- or CPP-BoNTA as the amount of proteins that, when intramuscularly injected into each mouse, result in 50% death.

### Evaluation of the *In Vivo* Potency of CPP-BoNTA Proteins

**FIGURE 5:**
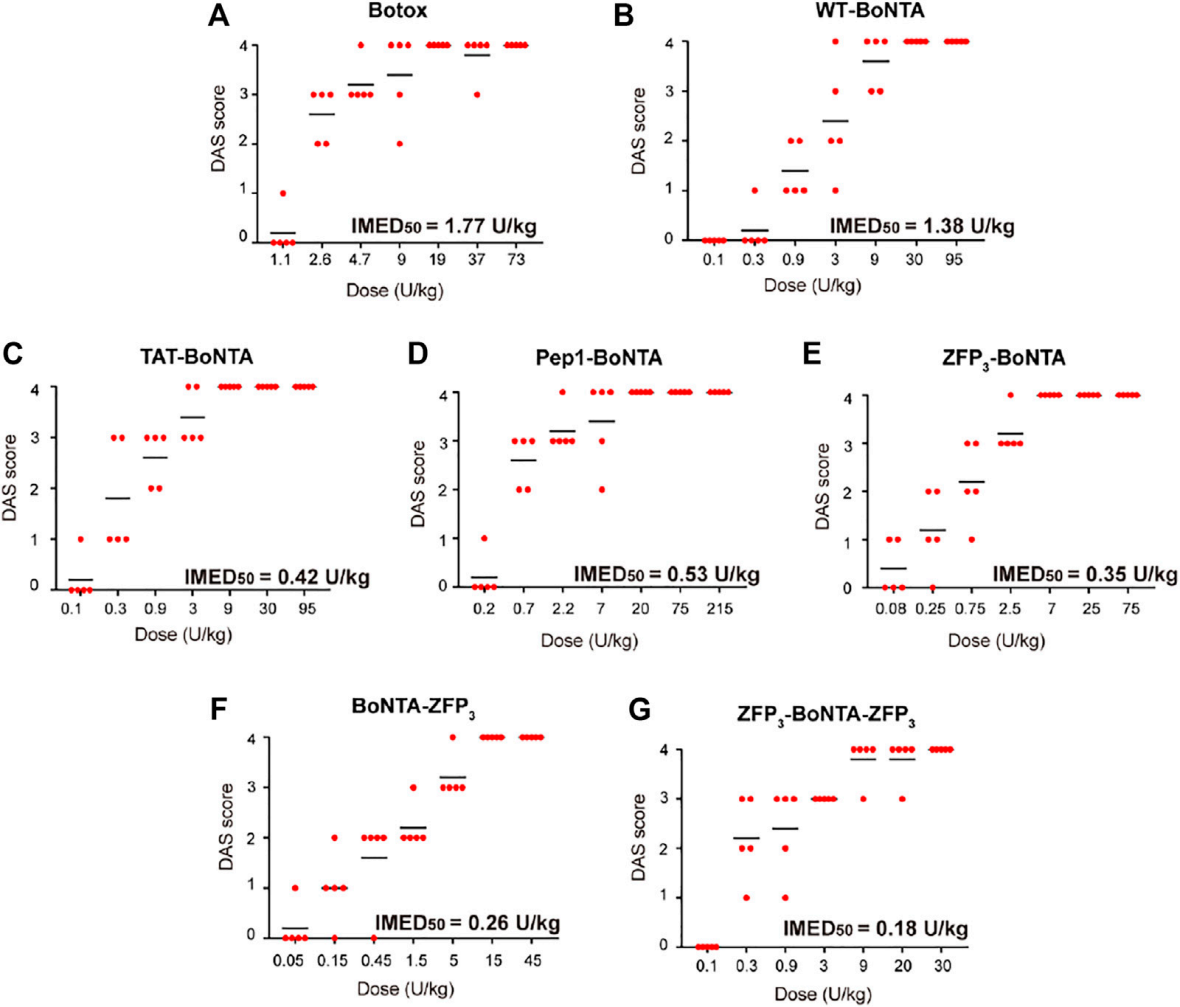
Evaluation of the *in vivo* potency of CPP-BoNTA proteins. Dose-dependent muscle-paralyzing activity of **(A)** Botox **(B)** BoNTA **(C)** TAT-BoNTA **(D)** Pep1-BoNTA **(E)** ZFP_3_-BoNTA **(F)** BoNTA-ZFP_3_ **(G)** ZFP_3_-BoNTA-ZFP_3_ as determined by DAS at day 2 after treatment.

The muscle-weakening effects of CPP-BoNTA, as determined by digit abduction score (DAS) assay (Aoki, 2001), were dose- and time-dependent with the peak values observed typically at day 2 after treatment (Supplementary Figure S3). We thus used DAS values at day 2 to determine the *in vivo* potency of WT- and CPP-BoNTA (Figures 5A–G). The IMED_50_ of BoNTA was defined as the amount of proteins that led to half of the mice exhibiting a minimum DAS value of 2 (Aoki, 2001). It was found that in the term of active units, CPP-BoNTA proteins had similar or higher potency compared to WT-BoNTA (similar or lower IMED_50_ values) with ZFP_3_-BoNTA-ZFP_3_ exhibiting highest intramuscular efficacy (lowest IMED_50_) (Figure 5).

### Determination of the Therapeutic Index

One interesting discovery in this study was that insect cell-derived WT-BoNTA protein had notable difference from marketed onabotulinumtoxinA (Botox) in pharmacological properties (Table 1). Most importantly, compared with Botox or WT-BoNTA, all CPP-BoNTA showed increased therapeutic index, as defined by the difference between IMLD_50_ and IMED_50_, with up to 10-fold improvement observed with ZFP_3_-BoNTA-ZFP_3_ (Table 1).

**TABLE 1:** The therapeutic index of CPP-BoNTA proteins.

Samples	IMLD_50_ (ng/kg)	Units (= ng)	IMED_50_ (ng/kg)	IMED_50_ (U/kg)	Therapeutic index IMLD_50_ (ng/kg)/IMED_50_ (ng/kg)
Botox	3.41	0.068	0.12	1.77	28
WT-BoNTA	79.85	1.60	2.20	1.38	36
TAT-BoNTA	26.48	0.53	0.22	0.42	120
Pep1-BoNTA	34.89	0.70	0.37	0.53	94
ZFP_3_-BoNTA	98.97	1.98	0.70	0.35	142
BoNTA- ZFP_3_	168.61	3.37	0.89	0.26	189
ZFP_3_-BoNTA -ZFP_3_	780.89	15.62	2.77	0.18	282

*Note*: In the present study, the active unit (U) of Botox is re-defined as described in the *Materials and Methods*. One unit of BoNTA, sample is defined as the amount of proteins that, when intramuscularly injected into each mouse, result in 50% death.

## Discussion

Native BoNTA contains HC and LC domains that are responsible for cellular translocation and target cleavage (Rossetto et al., 2014). In the present study, we used BV-Sf9 insect cells to express full-length BoNTA (Band et al., 2010). Insect cell-derived WT-BoNTA exhibited approximately 20-fold decrease in intramuscular toxicity compared with marketed Botox, as determined by IMLD_50_ (Table 1). In addition, other properties of recombinant WT-BoNTA including IMED_50_ and therapeutic index are different from those of marketed Botox. This discrepancy could arise from the accessory proteins in Botox, maturation or folding of proteins, or different formulation in the sample.

The CPPs used in this study contain human immunodeficiency virus (HIV)-derived Tat peptide (Fawell et al., 1994), synthetic Pep1 peptide (Morris et al., 2001) and Cys_2_-His_2_ zinc finger transcription factor-derived ZFP peptide (Gaj et al., 2014). Importantly, the ZFP CPP was derived from a mouse zinc finger Zif268 (Pavletich and Pabo, 1991) and has been engineered to abolish the DNA-binding activity (Gaj et al., 2014). Genetic fusion with CPP is often found to reduce the expression and purification efficiencies of cargo proteins (Liu et al., 2014). However, it appeared that in the present study all CPP-BoNTA fusion proteins could be acquired with high purity from the BV-Sf9 insect cell expression system though the yield may vary between different constructs. This particular feature has established insect cells an efficient platform for production of full-length BoNTAs and engineered derivatives.

Previous studies have suggested that ZFPs have higher cell-penetrating activity compared to conventional CPPs and can retain the activity of delivered cargo proteins (Gaj et al., 2014; Pang et al., 2019). It was surprising to find that N-terminal, C-terminal and bipartite ZFP fusions all reduced the *in vitro* protease activity of BoNTA whereas TAT and Pep1 did not compromise the activity (Figure 1C). It was possible that the reduced activity of BoNTA variants might result from the relatively larger sizes of ZFPs used in this study, where three tandem ZFP repeats were placed in an array (ZFP_3_). ZFP_3_ contains more than 90 amino acids which could create structural hindrance, in comparison with less than 10 amino acids in length in TAT or Pep1 fusion.

The internalization of BoNTA into mammalian cells relies on SV2 receptor. This receptor-mediated entry mechanism allows for cell-specific delivery of BoNTA for *in vivo* application and is an important reason for the notable safety of BoNTA products in clinical applications. It has been shown that ZFP penetrates cells through macropinocytosis and, to a lesser extent, caveolin-dependent endocytosis (Gaj et al., 2014). In addition, TAT or other CPPs may penetrate cells through water pore-mediated direct translocation (Trofimenko et al., 2021). Therefore, one may envision that fusion of CPPs can compromise the cell-targeting specificity of BoNTA proteins. However, our results have demonstrated that internalization of CPP-BoNTA is still SV2-dependent, as evidenced by the co-localization of delivered BoNTA and SV2 receptor when examined *in vitro* and *in vivo* (Figures 2, 3). This suggests that CPP fusion does not compromise the cell-type specificity of BoNTA but instead simply enhances the cellular uptake of BoNTA. This observation was surprising given that different CPPs have distinct mechanisms of cellular uptake. It would be thus interesting to investigate in future studies the fate of internalized BoNTA, such as subcellular localization.

One interesting discovery was that although all CPP fusion had minor or no effects on the *in vitro* cytotoxicity of BoNTA (Supplementary Figure S2A), different CPPs had various effects on the systemic toxicity of BoNTA in mice (Table 1). Specifically, TAT and Pep1 increased the *in vivo* toxicity of BoNTA with lower IMLD_50_ while all ZFP constructs, including N-, C- or bipartite fusion, had decreased *in vivo* toxicity. These suggested that ZFP might mechanistically be different from conventional CPPs, which requires more in-depth investigation in future studies.

Additionally, it was found that all recombinant BoNTA exhibited higher therapeutic index (TI) than marketed product Botox. Moreover, CPP fusion further increased the TI values with ZFP_3_-BoNTA-ZFP_3_ displaying more than 10-fold increase. We speculated that the increased TI values in CPP-BoNTA were related with the enhanced cellular uptake. It is possible that enhanced cellular uptake, particularly at the tissue level, could reduce the amount of intercellular BoNTA and, as a result, reduce the immune response associated with BoNTA. This possibility will be investigated in future studies.

In summary, in the present study we designed and characterized a series of CPP-BoNTA fusion proteins. Our study shed the light on improving the pharmacological properties of BoNTA using CPP fusion. We envision that this novel approach can be immediately expanded to other BoNT products for developing next-generation neuromuscular modulators.

## Data Availability

The original contributions presented in the study are included in the article/Supplementary Material, further inquiries can be directed to the corresponding author.
