# Supplementary material for: Cell-Penetrating Botulinum Neurotoxin Type A With Improved Cellular Uptake and Therapeutic Index
Source: Front Bioeng Biotechnol. 2022 Feb 11;10:828427. doi: 10.3389/fbioe.2022.828427 (PMC8874009; doi:10.3389/fbioe.2022.828427)
Supplement: Supplementary file 1 [file DataSheet1.docx]

Supplementary Material

## Supplementary Figures


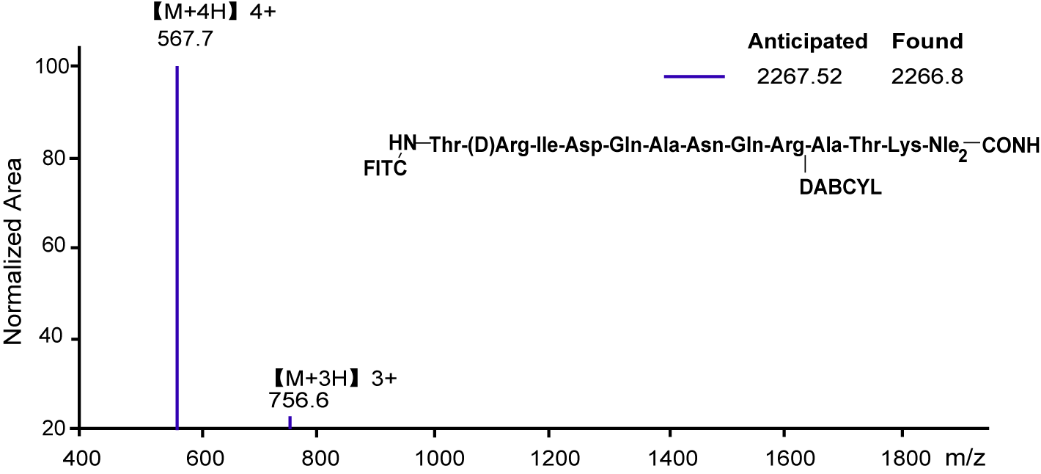


**Figure S1.** MS confirmation of synthesized FRET peptide reporter of BoNTA.


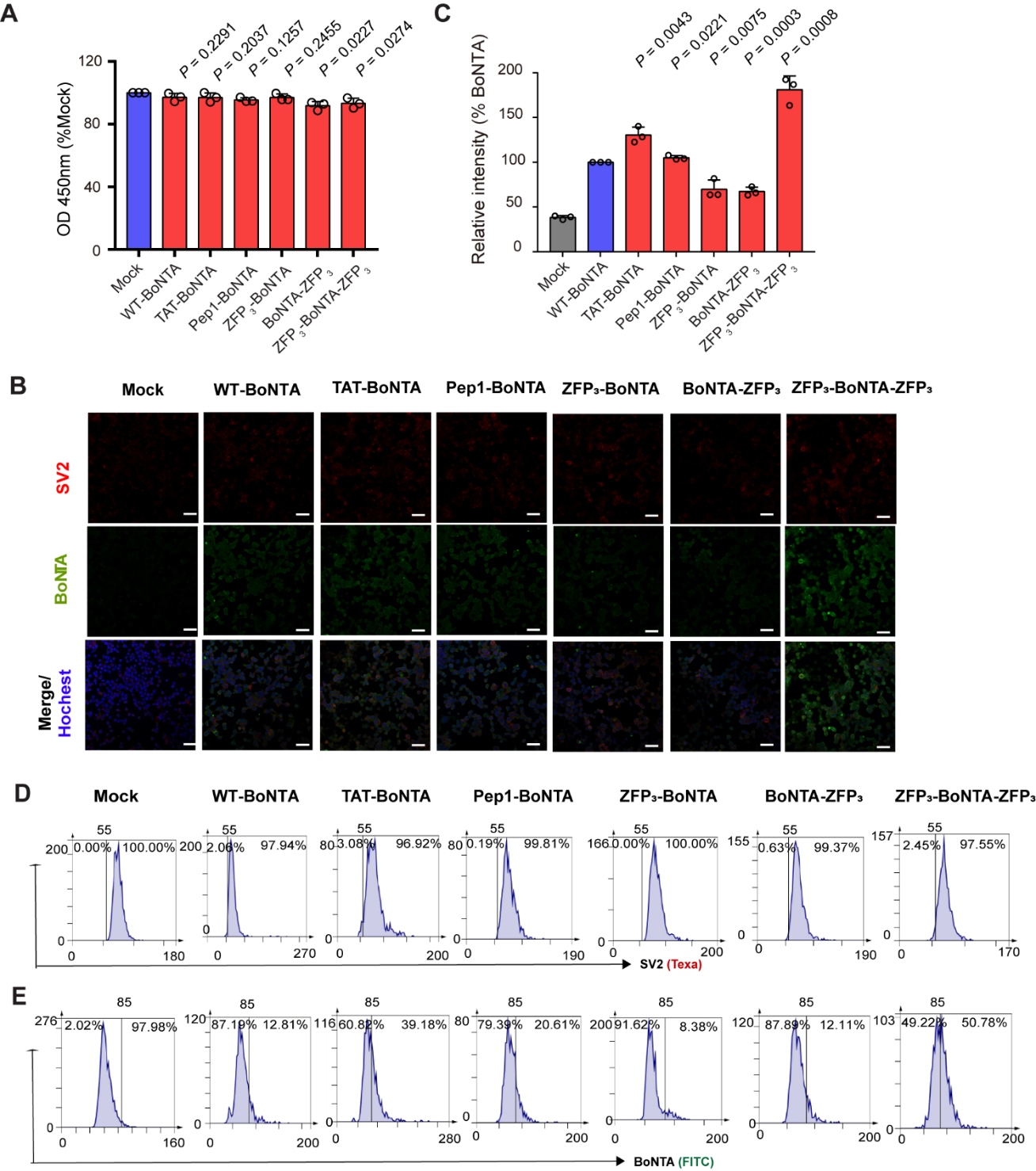


**Figure S2.** Evaluation of the cytotoxicity and cell-penetrating activities of BoNTA proteins in N2a cells. **(A)** The effects of CPP fusion on the cytotoxicity of BoNTA. **(B-C)** Immunofluorescence (IF) analysis of the cell-penetrating activity of CPP-BoNTA with 4 h incubation. **(B)** Representative images. Scale bars, 20 μm. **(C)** Quantification of the mean fluorescence intensity of BoNTA positive cells. **(D-E)** Histogram showing TissueFAXS cytometry analyses of co-localization of BoNTA proteins and SV2 receptors in mouse N2a cells (Related to Figure 2C). SV2 **(D)** and BoNTA **(E)** are analyzed in Texas Red and FITC channels respectively. Three biological replicates are performed and 1,000 cells are analyzed for each replicate. **(A and C)** The data are shown as mean ± SD (*n* = 3). Statistical analysis is performed using Student’s *t* test.


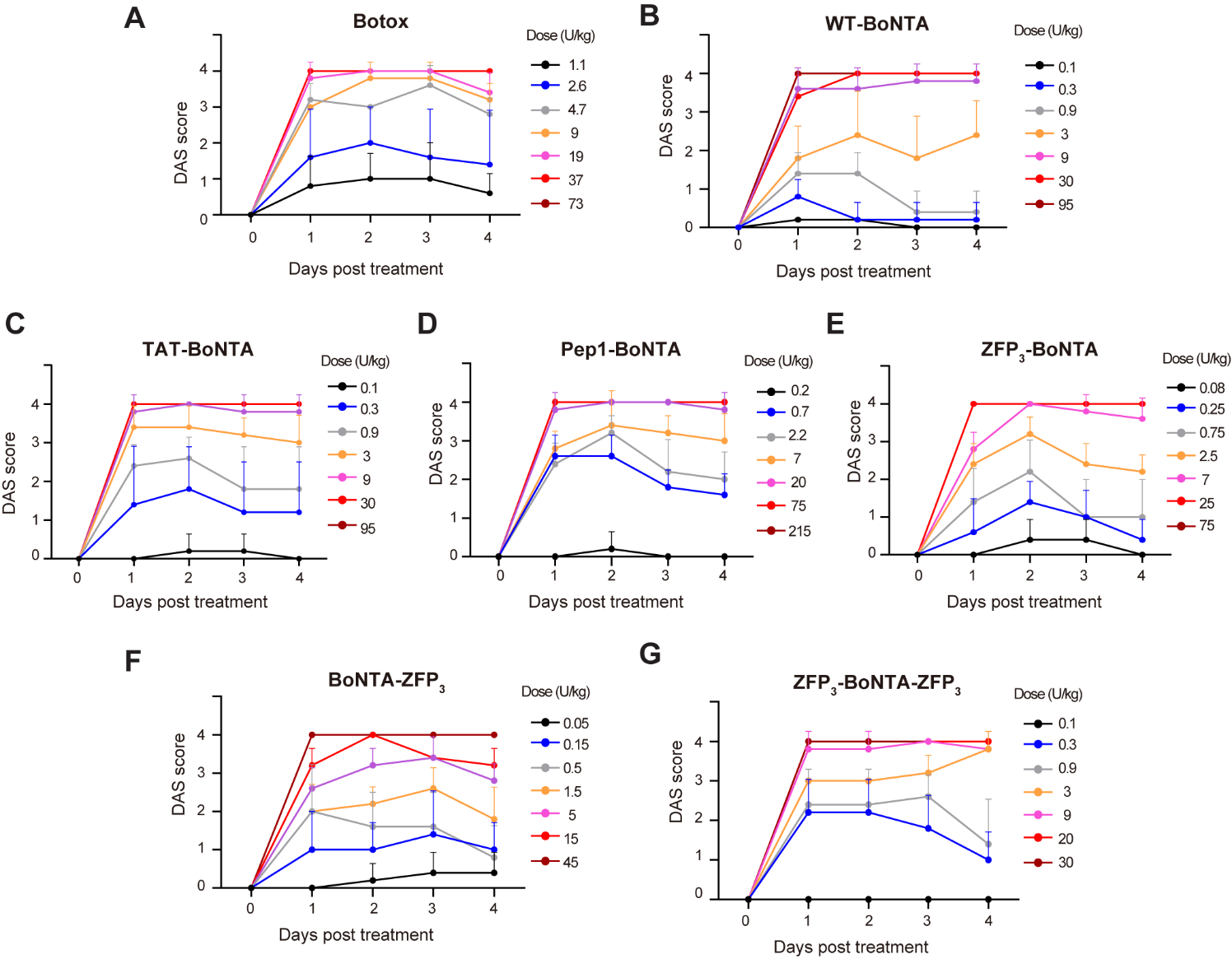


**Figure S3.** Analyses of time-dependent muscle-weakening effects of **(A)** Botox **(B)** BoNTA **(C)** TAT-BoNTA **(D)** Pep1-BoNTA **(E)** ZFP_3_-BoNTA **(F)** BoNTA-ZFP_3_ **(G)** ZFP_3_-BoNTA-ZFP_3_ using DAS assay.

## Supplementary Table

**Supplementary Table 1.** Primer sequences for CPP-BoNTA cloning.

| Primer name | Sequence ( 5’ to 3’ ) |
| --- | --- |
| BoNTA-FWD | gcgcgtctagaatggattataaggatgacgacgacaagggttccggttcccatcatcaccaccatcacagttccggcgtggacctgggtttcgagaacctgtacttccagggtatgcctttcgttaataaa |
| BoNTA-REV | atataaagcttttacagagggcgttcgcccca |
| TAT-BoNTA-FWD | gcgcgtctagaatggattataaggatgacgacgacaagggttccggttcccatcatcaccaccatcacagttccggcgtggacctgggtttcgagaacctgtacttccagggtggtcgtaaaaaacgccgc |
| TAT-BoNTA-REV | atataaagcttttacagagggcgttcgcccca |
| Pep1-BoNTA-FWD | gcgcgtctagaatggattataaggatgacgacgacaagggttccggttcccatcatcaccaccatcacagttccggcgtggacctgggtttcgagaacctgtacttccagggtaaagagacctggtgggaa |
| Pep1-BoNTA-REV | atataaagcttttacagagggcgttcgcccca |
| ZFP_3_-BoNTA-FWD | gcgcgtctagaatggattataaggatgacgacgacaagggttccggttcccatcatcaccaccatcacagttccggcgtggacctgggtttcgagaacctgtacttccagggtgaaaaaccgtacaagtgc |
| ZFP_3_-BoNTA-REV | atataaagcttttacagagggcgttcgcccca |
| BoNTA- ZFP_3_-FWD | gcgcgtctagaatgccttttgtgaacaagca |
| BoNTA- ZFP_3_-REV | gcgcgaagcttcttgtcgtcgtcatccttataatccatggaaccggaaccgtgatggtggtgatgatggaaacccaggtccacgccggaactaccctggaagtacaggttctcgccggtatgggtacgttg |
| ZFP_3_-BoNTA-ZFP_3_-FWD | gcgcgtctagaatggattataaggatgacgacgacaagggttccggttcccatcatcaccaccatcacagttccggcgtggacctgggtttcgagaacctgtacttccagggtgagaagccgtataaatgcc |
| ZFP_3_-BoNTA- ZFP_3_-REV | agtacttctcgacaagcttagccggtatgggtacgttggtgtgcc |

# 3 Supplementary Data

**Protein sequence**

**BoNTA full-length sequence (BoNTAFL)**

MPFVNKQFNYKDPVNGVDIAYIKIPNVGQMQPVKAFKIHNKIWVIPERDTFTNPEEGDLNPPPEAKQVPVSYYDSTYLSTDNEKDNYLKGVTKLFERIYSTDLGRMLLTSIVRGIPFWGGSTIDTELKVIDTNCINVIQPDGSYRSEELNLVIIGPSADIIQFECKSFGHEVLNLTRNGYGSTQYIRFSPDFTFGFEESLEVDTNPLLGAGKFATDPAVTLAHELIHAGHRLYGIAINPNRVFKVNTNAYYEMSGLEVSFEELRTFGGHDAKFIDSLQENEFRLYYYNKFKDIASTLNKAKSIVGTTASLQYMKNVFKEKYLLSEDTSGKFSVDKLKFDKLYKMLTEIYTEDNFVKFFKVLNRKTYLNFDKAVFKINIVPKVNYTIYDGFNLRNTNLAANFNGQNTEINNMNFTKLKNFTGLFEFYKLLCVRGIITSKTKSLDKGYNKALNDLCIKVNNWDLFFSPSEDNFTNDLNKGEEITSDTNIEAAEENISLDLIQQYYLTFNFDNEPENISIENLSSDIIGQLELMPNIERFPNGKKYELDKYTMFHYLRAQEFEHGKSRIALTNSVNEALLNPSRVYTFFSSDYVKKVNKATEAAMFLGWVEQLVYDFTDETSEVSTTDKIADITIIIPYIGPALNIGNMLYKDDFVGALIFSGAVILLEFIPEIAIPVLGTFALVSYIANKVLTVQTIDNALSKRNEKWDEVYKYIVTNWLAKVNTQIDLIRKKMKEALENQAEATKAIINYQYNQYTEEEKNNINFNIDDLSSKLNESINKAMININKFLNQCSVSYLMNSMIPYGVKRLEDFDASLKDALLKYIYDNRGTLIGQVDRLKDKVNNTLSTDIPFQLSKYVDNQRLLSTFTEYIKNIINTSILNLRYESNHLIDLSRYASKINIGSKVNFDPIDKNQIQLFNLESSKIEVILKNAIVYNSMYENFSTSFWIRIPKYFNSISLNNEYTIINCMENNSGWKVSLNYGEIIWTLQDTQEIKQRVVFKYSQMINISDYINRWIFVTITNNRLNNSKIYINGRLIDQKPISNLGNIHASNNIMFKLDGCRDTHRYIWIKYFNLFDKELNEKEIKDLYDNQSNSGILKDFWGDYLQYDKPYYMLNLYDPNKYVDVNNVGIRGYMYLKGPRGSVMTTNIYLNSSLYRGTKFIIKKYASGNKDNIVRNNDRVYINVVVKNKEYRLATNASQAGVEKILSALEIPDVGNLSQVVVMKSKNDQGITNKCKMNLQDNNGNDIGFIGFHQFNNIAKLVASNWYNRQIERSSRTLGCSWEFIPVDDGWGERPL

**WT-BoNTA (His6-BoNTAFL)**

MDYKDDDDKGSGS**HHHHHH**SSGVDLGFENLYFQG-BoNTAFL

**TAT-BoNTA (His6-TAT-BoNTAFL)**

MDYKDDDDKGSGS**HHHHHH**SSGVDLGFENLYFQG**GRKKRRQRRRPQ**GGSGGSGGS-BoNTAFL

**Pep1-BoNTA (His6-Pep1-BoNTAFL)**

MDYKDDDDKGSGS**HHHHHH**SSGVDLGFENLYFQG**KETWWETWWTEWSQPKKKRKV**GGSGGSGGS-BoNTAFL

**ZFP_3_-BoNTA (His6-ZFP_3_-BoNTAFL)**

MDYKDDDDKGSGS**HHHHHH**SSGVDLGFENLYFQG**EKPYKCPECGKSFSASAALVAHQRTHTGEKPYKCPECGKSFSASAALVAHQRTHTGEKPYKCPECGKSFSASAALVAHQRTHTG**GGSGGSGGS-BoNTAFL

**BoNTA-ZFP_3_ (BoNTAFL-ZFP_3_-His6)**

BoNTAFL-GGSGGSGGS**EKPYKCPECGKSFSASAALVAHQRTHTGEKPYKCPECGKSFSASAALVAHQRTHTGEKPYKCPECGKSFSASAALVAHQRTHTG**ENLYFQGSSGVDLGF**HHHHHH**GSGSMDYKDDDDK

**ZFP_3_-BoNTA-ZFP_3_ (His6-ZFP_3_-BoNTAFL-ZFP_3_)**

MDYKDDDDKGSGS**HHHHHH**SSGVDLGFENLYFQG**EKPYKCPECGKSFSASAALVAHQRTHTGEKPYKCPECGKSFSASAALVAHQRTHTGEKPYKCPECGKSFSASAALVAHQRTHTG**GGSGGSGGS-BoNTAFL-GGSGGSGGS**EKPYKCPECGKSFSASAALVAHQRTHTGEKP YKCPECGKSFSASAALVAHQRTHTGEKPYKCPECGKSFSASAALVAHQRTHTG**
